# Assessing the effect of mHealth on child feeding practice in African countries: systematic and meta-analysis

**DOI:** 10.1186/s41043-023-00487-y

**Published:** 2023-12-08

**Authors:** Girma Gilano, Sewunet Sako, Temesgen Dileba, Andre Dekker, Rianne Fijten

**Affiliations:** 1 Department of Public Health Informatics, School of Public Health, College of Medicine and Health Sciences, Arba Minch University, P.O Box: 21, Arba Minch, Ethiopia; 2 Department of Radiation Oncology [Maastro], GROW School for Oncology and Developmental Biology, Maastricht University Medical Centre+, Mastro Clinic, P.O. Box 616, 6200 MD Maastricht, The Netherlands

**Keywords:** mHealth, Child feeding, Meta-analysis, Africa

## Abstract

**Introduction:**

Poor child feeding practice is a public health problem in Africa. Mobile health (mHealth) is a supportive intervention to improve this problem; however, the evidence available in the current literature is inconsistent and inconclusive in Africa. Some studies state that exclusive breastfeeding is not different between controls and mHealth interventions in the first month. Other studies state that health providers need additional training for the success of mHealth interventions.

**Objective:**

This systematic review and meta-analysis aims to provide the summarized effect of mHealth on child-feeding practices in Africa to improve future planning and decisions.

**Method:**

We conducted a systematic review and meta-analysis based on the published and unpublished evidence gathered from PubMed, Web of Science, Cochrane Library, and Embase databases between January 1, 2000, and March 1, 2022. Studies included were randomized control trials and experimental studies that compared mHealth to standards of care among postpartum women. Preferred Reporting Items for Systematic Review and Meta-analysis guidelines followed for the reporting.

**Results:**

After screening 1188 studies, we identified six studies that fulfilled the study criteria. These studies had 2913 participants with the number of total intervention groups 1627 [1627/2913 = 56%]. Five studies were completed within 24 weeks while one required 12 weeks. We included two RCTs, two cluster RCTs, and two quasi-experimental studies all used mHealth as the major intervention and usual care as controls. We found significant improvement in child-feeding practices among intervention groups.

**Conclusion:**

This systematic review and meta-analysis showed that the application of mHealth improved child-feeding practices in Africa. Although the finding is compelling, the authors recommend high-quality studies and mHealth interventions that consider sample size, design, regional differences, and environmental constraints to enhance policy decisions. The place of residence, access, low socioeconomic development, poor socio-demographic characteristics, low women empowerment, and low women’s education might cause high heterogeneity in the included regions and need consideration during interventions.

*Registration number*: PROSPERO: CRD42022346950.

**Supplementary Information:**

The online version contains supplementary material available at 10.1186/s41043-023-00487-y.

## Introduction

Over the last few decades, child-feeding practice has been a prominent public health problem in Africa [1]. Evidence shows that mobile health can improve the problem [2–5]. Mobile health is defined broadly as an emerging mobile phone-based communication technology used to improve access to healthcare. The devices involved may include mobile phones, patient monitoring devices, personal digital assistants, and any other wireless devices used to improve access to health information and to support the achievement of health objectives [6]. According to the United Nations Children’s Fund [UNICEF], technologies are important because of economic, political, market, social, and cultural challenges [7]. United Nations [UN] also reported 149 million stunted under five years old children, 45 million wasted, and 38.9 million over-weighted in 2020 that can be improved through mHealth [8, 9]. Mobile health technology improves child feeding practices, cord care, thermal care, delayed bathing of babies, safer sleep practices, care-seeking, and problem-solving during the postnatal period in rural areas in Africa [10]. Mobile health promotes maternal education and knowledge of child-feeding practices [11]. It enables a substantial number of families to follow child-feeding guidelines in low and middle-income countries [12] causing 83% adherence to dietary guidelines [13].

Some studies showed that mHealth-based counseling is substantially supported in communities of various cultural beliefs and socioeconomic status [14, 15] and sustains a high rate of exclusive breastfeeding [EBF] [16–18]. For example, short message service (SMS) delayed the time for complementary feeding, increased awareness of the World Health Organization child feeding guidelines, and improved maternal knowledge on child feeding in countries of different cultures [19–21]. This may illustrate the improved maternal knowledge regarding child nutrition in various cultural background areas [22]. For instance, mHealth improved the rate and duration of EBF in Nigeria [17] and improved initiation of breastfeeding after birth in Uganda [23]. Mobile health information enables safe breastfeeding for women with various health problems [24]. It enhances EBF practices and early contraceptive use among mothers [25]. Mobile health can be used in groups [shared among family members or near neighbors] and can have an effect on individuals’ or groups’ knowledge of breastfeeding and complementary feeding according to the WHO schedule [26–29].

There are many modes of mHealth message delivery systems such as cell phone-based messaging, group counseling using common songs and dramas [30], and visual interaction of feeding practice [video-based mHealth] [31–33].

Many challenges identified through various studies were related to ownership of devices, internet access [34], types of phones to accommodate the varieties of messages [35], and lack of funds in low and middle-income countries including Africa [36]. Additionally, language barriers, literacy, education level, cultural aspects, health-seeking behavior, lack of standardization, lack of a regulatory framework, and health system readiness were other challenges [6, 23, 37–40].

Overall, the challenges cause inconclusive evidence throughout the continent. For instance, some studies showed EBF is not different between controls and mHealth intervention groups in the first month [23, 36, 37] and others stated the need for health providers’ support in additional mHealth intervention [30, 41, 42]. Although mHealth alone can provide successful improvement in child feeding practice [15, 43, 44], there is still an argument to introduce additional interventions with mHealth [41]. Thus, this review aims to summarize the evidence and provide conclusive evidence for policy and decision-making.

### Objectives

To summarize evidence on the effects of mHealth on child-feeding practices in African countries.

## Methods

### Study protocol

The study protocol was approved by the AMU-IRB research review committee [AMU-IRB/1316/2022] on the 18th of July, 2022.

### Research question

We described the research question based on the population, intervention, comparator, and outcome [PICO] criteria as follows.

Is mHealth an effective alternative to standard care to improve child-feeding practices in Africa?

### Study design

A systematic review and meta-analysis of all randomized trials, interventional, longitudinal, and population cohort studies that used mobile phones in child-feeding practices in comparison to usual care.

### Inclusion

1. Studies conducted between January 1, 2000, to March 1, 2022, to include more mHealth studies from the time intervention started to the recent.
2. Conducted on a population of pregnant or postpartum mothers.
3. Randomized trials or interventional and longitudinal population cohort studies reported in the English language.
4. Conducted using an intervention that involves mHealth [phone calls, voice messages, text messages, interactive computer system and others].
5. Conducted with a primary endpoint of the timing of breastfeeding practice at different months.
6. Conducted with controls referred to Standards or usual Care.

### Exclusion criteria

1. Studies with poor methodological quality or difficulty fitting into the local context.
2. Studies with clear initial differences between interventions and controls (if studies have a dis-similar population in each group).
3. Studies for which results cannot be obtained.
4. Studies reporting in non-English languages [cannot obtain translation].

### Information source

We applied different search strategies to find available resources. Since our study includes both published and unpublished literature, we used PubMed, Cochrane Library, Web of Science, EMBASE, ClinicalTrials.gov, Sciencedirect, African Journals Online (AJOL), and WHO International Clinical Trials Registry Platform (ICTRP) [37].

*Key Search terms*: “Cell Phone*”, “Handheld Computer*”, “Multimedia*”, “Smartphone*”, “Technology Addiction”, “Cell Phone Use*”, “Telephone*”, “Text Messag*”, “Mobile App*”, “Patient Portal*”, “Internet-Based Intervention*”, “Hotline*”, “Telemedicine*”, “MP3-Player*”, “Webcasts as Topic*”, “Webcast*”, “Biomedical Assessment Technolog*”, “Biomedical Technolog*”, “Medical Informatics”, “Public Health Informatics”, “Marketing of Health Service*”, “Multimedia*”, “Wireless Technolog*”, “Electronic Mail*”, “Internet*” “Prenatal Care”, “Postnatal Care”, “Child Health Services”, “Maternal-Child Health Services” “Immunization Programs”, ( “Vaccination Coverage”, “Vaccination”.

### Search strategies

We provided the search strategy for eight databases elsewhere [Additional file 1].

The Preferred Reporting Items for Systematic Reviews and Meta-Analyses [PRISMA] guideline was used to preform activities.

### Study selection, quality appraisal, and data extraction

We searched studies from the designated databases and exported them to Endnote X20 to remove duplicate files. Two individuals [SS and TD] screened the rest of the articles after removing duplicates where title, abstract, and full-text appraisal were conducted subsequently. A third party settled the disagreement between reviewers. This means the two reviewers may not agree on some criteria about the observed characteristics of a given article during screening. Thus, another person is required to assist them in resolving the disagreement [GG]. We also checked the quality of the studies using the Joanna Briggs Institute [JBI] critical appraisal checklist trial studies [45]. In JBI, each study uses the 13 criteria checklist with scores extending from 0 to 13. The tool contains yes, no, unclear, and not applicable responses. The score yes is 1 and 0 for all others. The higher the score the lower the risk of bias. Two independent individuals [SS and TD] reviewed the retained articles before inclusion in the final review. Studies included in the final stage required to have a quality score of fifty or above. We also calculated the quality scores using the proportion of Cochrane criteria for bias assessment for each included article. We used Microsoft Excel for data capture and used the author's name, year of publication, year of study, study design, study area, response rate, sample size, study quality score, participants, setting, and duration to extract the data.

### Statistical methods and analysis

The Revman software with version 5.4.1 and comprehensive meta-analysis [CMA] prediction interval [46] were used for the analysis of this study. Forest plots were used to present the magnitude of change in child feeding due to the mHealth messaging application compared to the usual care in Africa. We assessed the heterogeneity and quantified I^2^ and τ^2^ statistics [between studies variances] and applied a *p* value of less than 0.05 to assume the presence of an association [47]. The I^2^ statistic is the percentage of variation due to heterogeneity not by chance across studies. I^2^ is intuitive and a simple expression of heterogeneity among studies. It is the percentage of variance attributable to study heterogeneity but cannot tell us the actual heterogeneity. There is no way to know the percentage of what it reports; it is just a percentage of an unknown number. However, in this study, we reported the prediction interval of the real effect size distribution in a comparable population.

To select a statistical model appropriate to a given review, it is important to consider where studies are coming from. One can consider a fixed effect model when all studies are coming from a fixed population effect size; for instance, a review based on the students’ Mathematics scores in a given school. In contrast, a random effect size model is considered when effect sizes are sampled from a population of universal effect sizes. We assumed a random effect model as our studies consider all regions in the African continent.

The τ^2^ is the heterogeneity variance whose square root is equivalent to the standard deviation [SD]. When τ^2^ is zero or smaller, the I^2^ becomes smaller which indicates uniformity across the studies. Generally, I^2^ is the proportion of errors due to effect size variation across studies plus sampling error while τ^2^ is a variation due to sampling error. We conducted a subgroup analysis by considering different study characteristics such as sample size [large or small], study regions [Eastern, Western, or Southern Africa], intervention [mHealth only or mHealth plus additional support], and study design [RCT, cluster RCT or quasi] to account for the variabilities [48].

We used the Egger regression asymmetry test and the Cochrane Collaboration Risk of Bias [CCRB] tool to check publication biases [49]. Egger’s test uses a funnel plot to assess potential publication biases in a meta-analysis. It is a linear regression test using standard errors weighted by their inverse variance. CCRB contains seven criteria: randomization, blinding participants, blinding assessment, allocation concealment, and select reporting. Based on the criteria we grade CCRB as high, low, or unclear risk of biases. We used a p-value of less than 0.05 to confirm the presence of publication bias. To estimate the number of missing studies from the meta-analysis, we also conducted Duval and Tweedie’s trim and fill method [50].

### Analyses of sensitivity

Sensitivity examinations were used to estimate the change in the selection of some studies with a risk of bias and those with a minimal difference. This is necessary to evaluate if the model statistical methods—random-effect and fixed-effect models cause a change in results and the changes that occur when some studies with a high risk of bias are excluded. The usual indicators of risk of bias in trial studies such as blinding outcome assessment, allocation concealment, and losses to follow-up [not greater than 25%] were considered [51]. Depending on how much effect a given study contributes to the total effect size, it is possible to know which study has more influence. By simply adding or removing studies stepwise from a group of studies, we easily identified the change in the pooled effect. The influence of a given study may arise from specific characteristics of that study and we can remove or keep a given study to have a more stable pooled effect size.

## Results

**Fig. 1:**
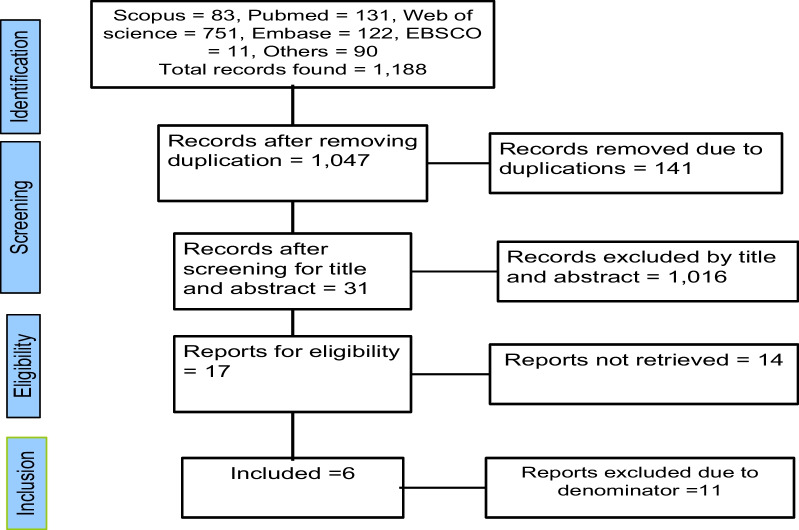
The PRISMA diagram showing the procedure followed during the systematic review meta-analysis screening for child feeding practices

Overall, we found 1188 articles related to child-feeding practices. After further screening, six articles remained that fulfilled the eligibility criteria. There is no study on prelacteal feeding, complementary feeding, and the timing of complementary feeding in Africa [Fig. 1].

### Characteristics of the included studies

All six included articles were breastfeeding-based interventional studies [17, 30, 41, 42, 52, 53]. Two of them were RCTs [17, 53], two cluster RCTs [30, 41], and two quasi-experimental [42, 52] studies. Overall, all six studies were completed within 24 weeks while one required only 12 weeks. All studies are within the period ranging from 2014 to 2022. Five of the studies were published and one was not published [QUT eprints] [52]. The mobile-based intervention was mostly text messages with some video and discussions initiated by either voice or video calls also included [52] [Table 1].

**Table 1:** Characteristics of the included studies in a systematic review and meta-analysis on child feeding practices in Africa

First authors, year	Type of intervention	Country	Intervention sample	Control sample	Attrition	Study design	Setting	Participants	Duration of intervention	Quality score (%)
Daprim, 2021 [13]	Mobile call- based support	Nigeria	67	64	12%	RCT	Public Hospital	Women	24 Weeks	86
Gebremariam, 2020 [38]	SMS text	Ethiopia	88	40	4.68%	Quasi-experimental research	Health Center	Women and partner	12 Weeks	57
Ogura, 2017 [13]	SMS text	Kenya	100	40	0.67%	RCT	A Public Sector Maternal Child Health [MCH]	Women + provider	24 Weeks	57
Flax. 2022 [39]	WhatsApp chat and text	Nigeria	600	600	7%	Quasi-experimental research[CRT]	Private Health Facilities	Women + provider	24 Weeks	71
Flax, 2014 [24]	Call, video, discussion	Nigeria	196	194	15%	Cluster RCT	Community	Group of women	24 Weeks	71
Adam, 2021 (39)	Video message	South Africa	747	755	1%	cluster-RCT	Nongovernmental Community Health Organization	Women	24 Weeks	71

### Assessing the risk of bias

**Fig. 2:**
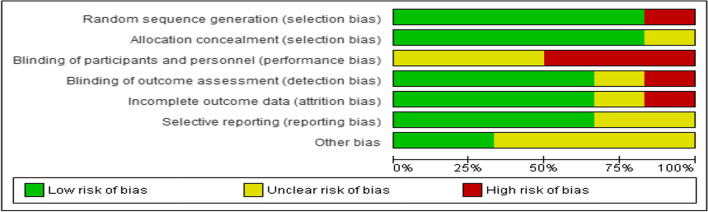
Risk of bias chart using each risk of bias item presented as percentages across all included studies

**Fig. 3:**
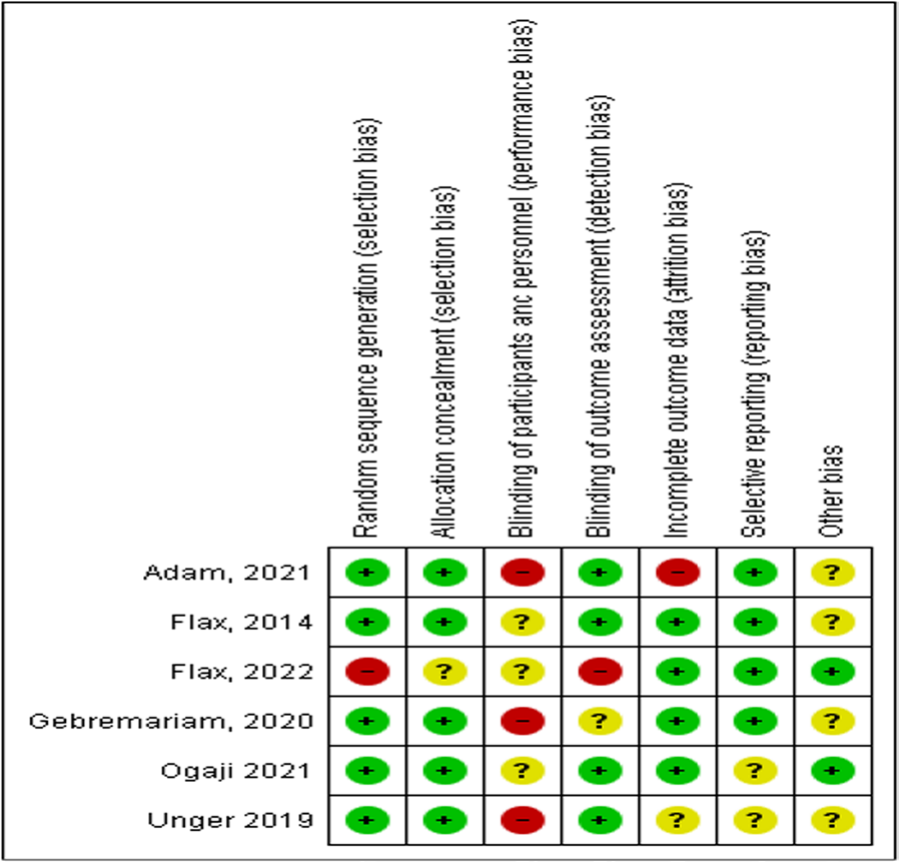
Risk of bias summary chart: review authors' judgments about each risk of bias item for each included study in a systematic review and meta-analysis on child feeding practices in Africa

Using the CCRB, all included studies showed quality scores of over 50%; however, some studies missed the clear presentation of random sequence generation [17, 52], blinding of participants and outcome assessment [52, 53], and other biases. From the included studies, one study does not mention random sequence generation during recruitment and two studies have no clear description of this procedure. Two studies do not mention blinding participants. Similarly, one study has no information on blinding data collectors, and another two have unclear descriptions. Overall, a moderate risk of bias was observed, which might slightly affect the findings of the study. We interpreted the results accordingly and recommended usage in light of these limitations [Figs. 2, 3, Table 2].

**Table 2:** The risk of biased assessed using Cochrane Risk of bias assessment tool

Cochrane scale	Ogaji, 2021	Gebremariam, 2020	Unger, 2019	Flax, 2022	Flax, 2014	Adam, 2021
Random sequence generation (Selection bias)	Yes	?	Yes	No	Yes	?
Allocation concealment (Selection bias)	Yes	Yes	Yes	Yes	Yes	Yes
Blinding of participants and personnel (Performance bias)	Yes	No	No	Yes	?	Yes
Blinding of outcome assessment (Detection bias)	Yes	?	Yes	No	Yes	?
Incomplete outcome data (Attrition bias)	No	Yes	Yes	Yes	Yes	Yes
Selective reporting (Reporting bias)	Yes	Yes	?	Yes	?	Yes
Other bias	Yes	Yes	?	**?**	?	?

### The effect of mHealth on child feeding practice

**Fig. 4:**
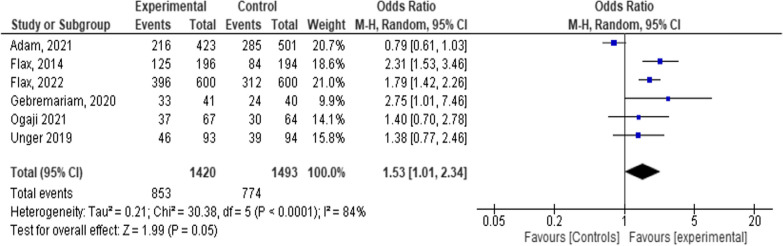
Forest plot depicting the relationship among six studies in a systematic review and meta-analysis on child feeding practices in Africa

Six studies with a sample size of 2,913 were included with total events of 1627 [1627/2913 = 56%]. The mean effect size based on the random-effects model was [OR = 1.53, 95% CI 1.01–2.34; *P* < 0.001; I^2^ = 84%]. This shows that mHealth has a 53% advantage over SOC [Fig. 4].

**Fig. 5:**
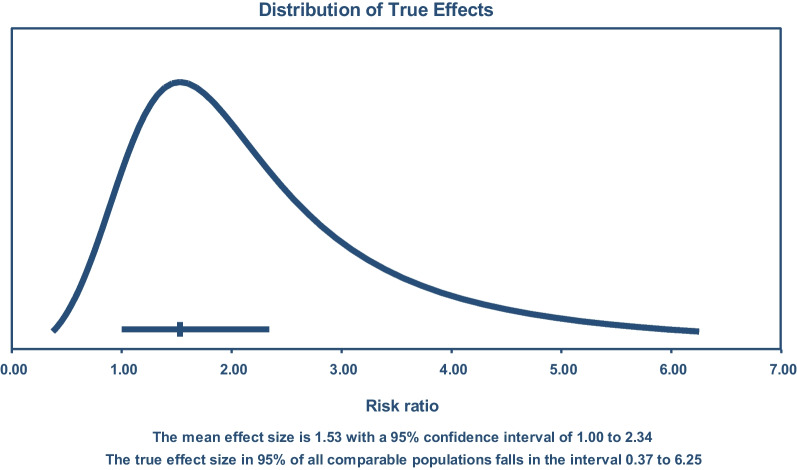
The distribution of true effect size among the comparable universal populations

From the mean effect size above, the I^2^ = 84% does not tell us the variation of effect size and the universal distribution of effect sizes. It indicates that 84% variation in effect size is true. Since we do not know the amount of variation of which 84% is true and there is no way to know that, we applied prediction interval software to show the distribution of true effect size. Thus, according to Fig. 5, the true effect size in 95% of all comparable populations falls in the interval of 0.37–6.25 unlike the I^2^ interval of 1.0–2.34 [Fig. 5]. In primary studies, we can apply standard deviation since we have only a sampling error when n > 30. In the meta-analysis, we have sampling error plus variation across each study [heterogeneity or I^2^]. Since we usually consider a small number of studies, we need to know at least the universal distribution of true effect size across all comparable populations [Fig. 5].

### Sensitivity test

**Fig. 6:**
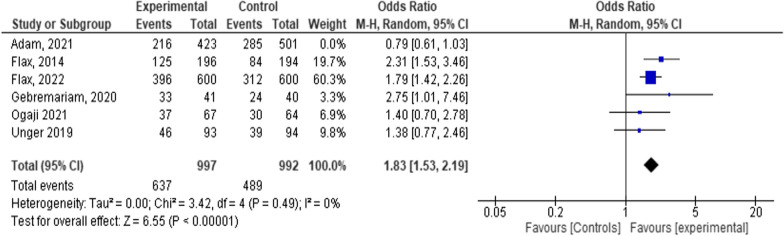
Forest plot depicting the overall result when one study weight is set to zero in a systematic review and meta-analysis on child feeding practices in Africa

From Fig. 4 above, we can understand that all observed variations [84%] were due to variations of true effect size. To know the influential study, we added or removed each study stepwise and checked the change that occurred in the mean effect size. We identified one study that caused substantial changes in the mean effect size when removed [41]. This study has lowered the overall effect size, and the extent of influence was assessed as follows. According to Cook's distances, none of the studies could be considered to be overly influential. Neither the rank correlation nor the regression test indicated any funnel plot asymmetry [*P* = 0.4694 and *P* = 0.1247, respectively]. For this reason, we preferred to keep all studies and maintained the pooled effect size of [OR = 1.53, 95% CI 1.01–2.34; *P* < 0.001; I^2^ = 84%] [Figs. 4, 6].

### Funnel plot

**Fig. 7:**
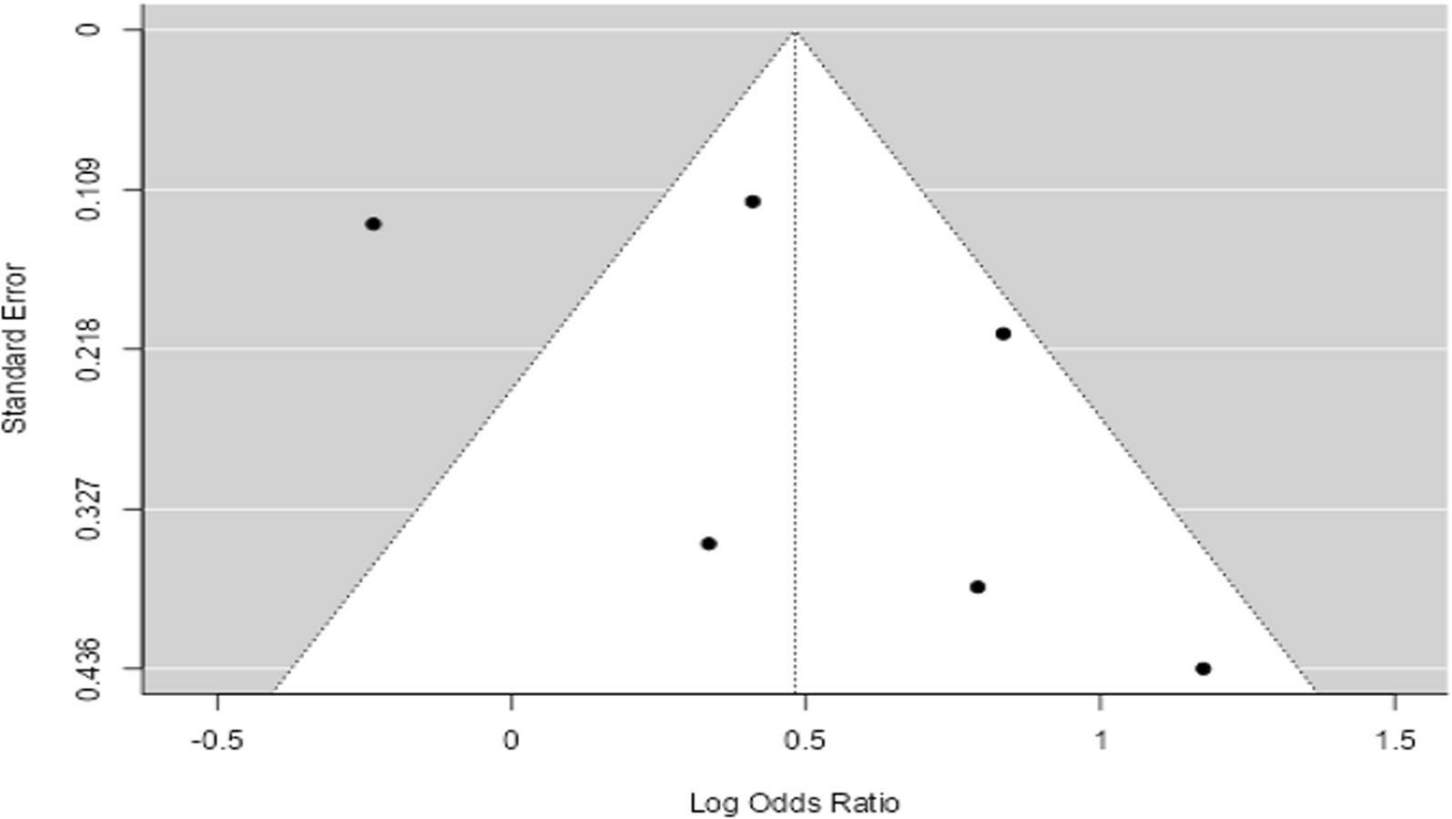
Funnel plot of comparison relationship among studies identify publication bias in a systematic review and meta-analysis on child feeding practices in Africa

According to the distribution of studies on the funnel plot below, there is no publication bias as the distribution is symmetric. Each dot on the figure represents a study; studies inside the white section are not statistically significant [no risk of bias] [Fig. 7].

## Subgroup analysis

### Analysis based on sample size and study designs

**Fig. 8:**
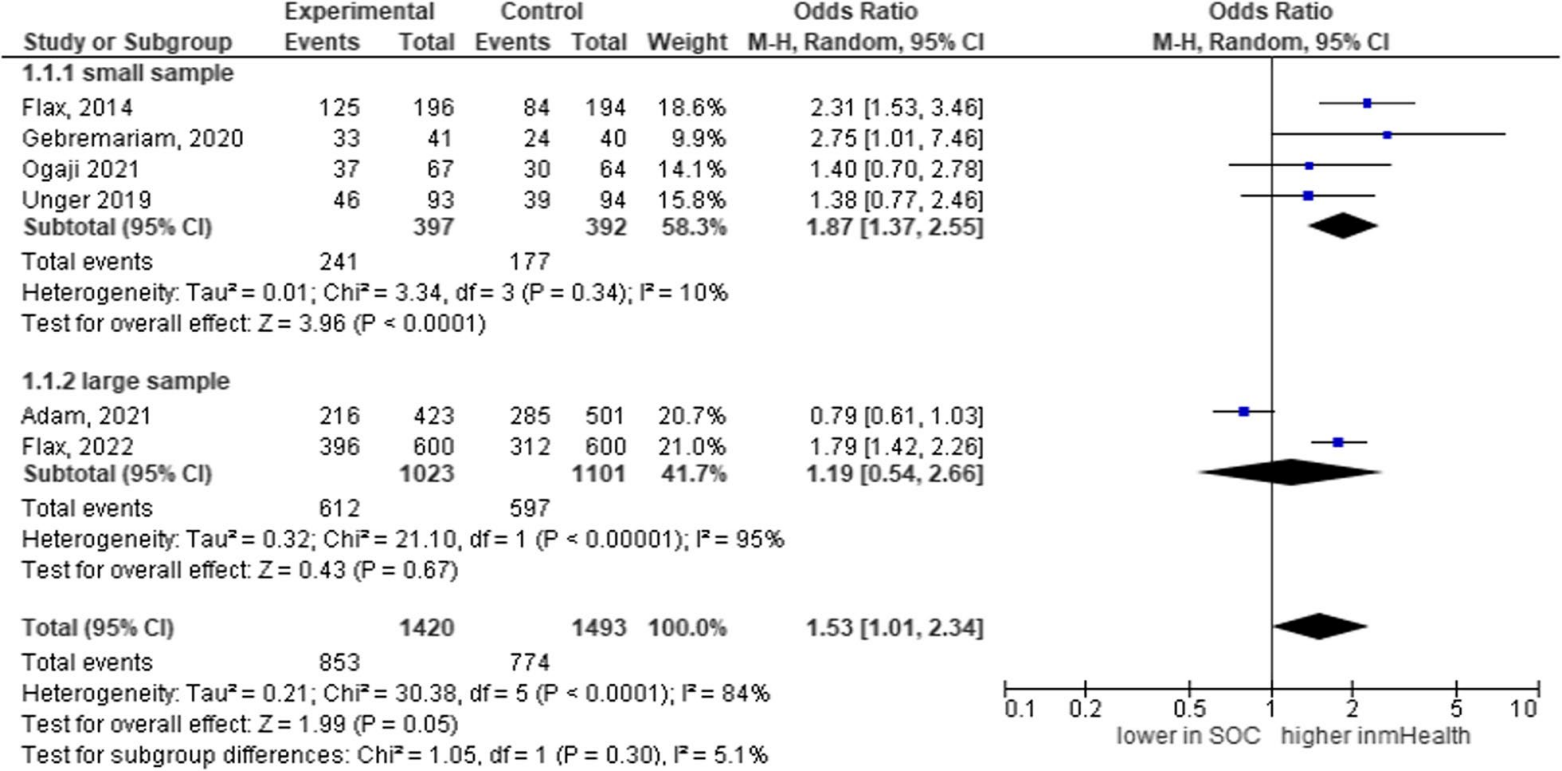
Forest plot depicting the subgroup analysis using the size of the study in a systematic review and meta-analysis on child feeding practices in Africa

From Fig. 8, the mean effect size of using mHealth was 53% [OR = 1.53, 95% CI 1.01, 2.34; *P* = 0.30; I^2^ = 5.1%]. With this observed heterogeneity, there is not enough evidence to say the studies in each group are different. The observed heterogeneity is only by chance and does not affect the interpretation of the findings.

**Fig. 9:**
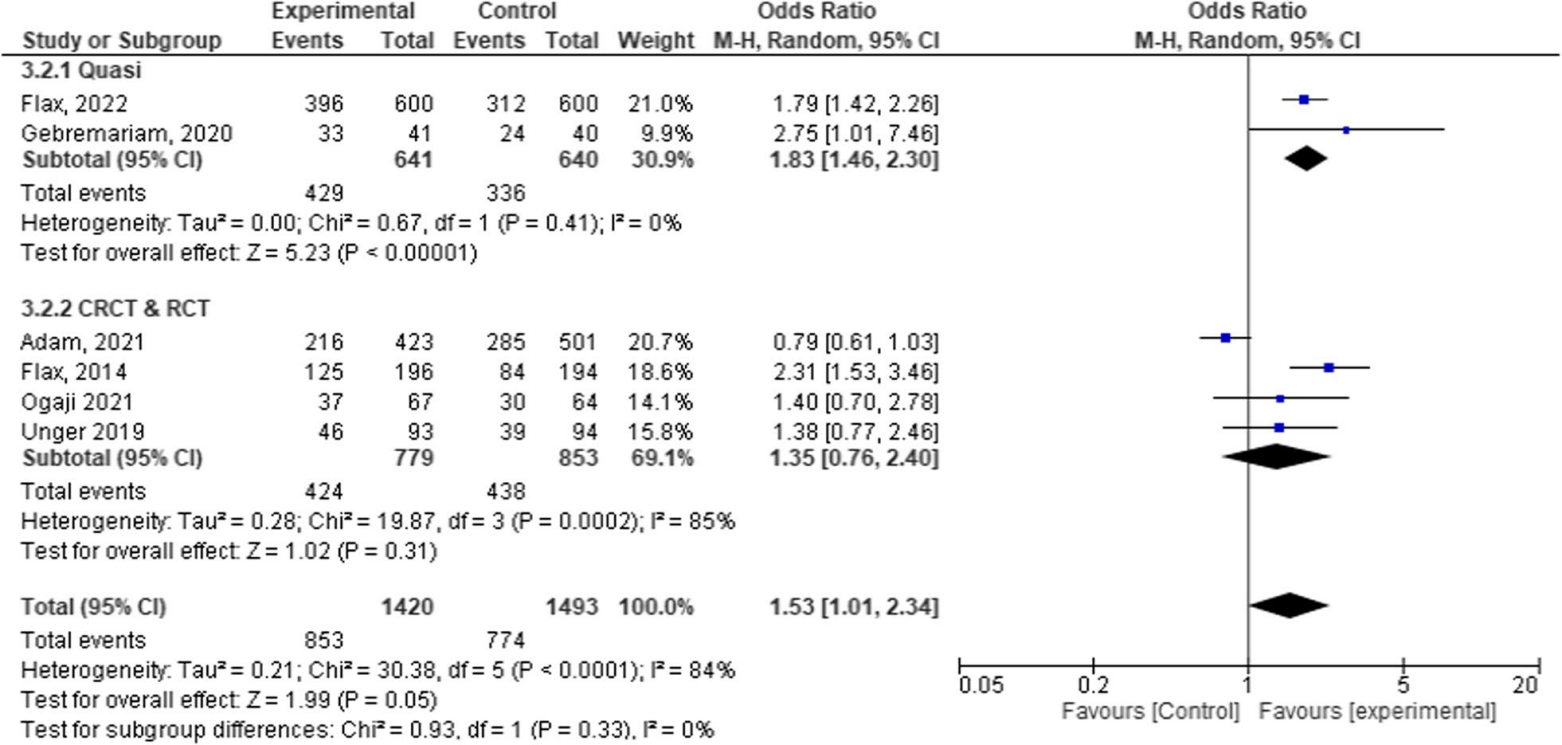
Forest plot of comparison of the effect of design differences among groups in a systematic review and meta-analysis on child feeding practices in Africa

From Fig. 9, the mean effect size of quasi-experiments and cluster or individual randomized trials was 53% [OR = 1.53, 95% CI 1.01, 2.34; *P* = 0.33; I^2^ = 0%]. This means the mHealth interventions using quasi-experiments, cluster randomized trials, and RCT studies showed uniform effect size on child feeding practice [Fig. 9].

### Regional differences

**Fig. 10:**
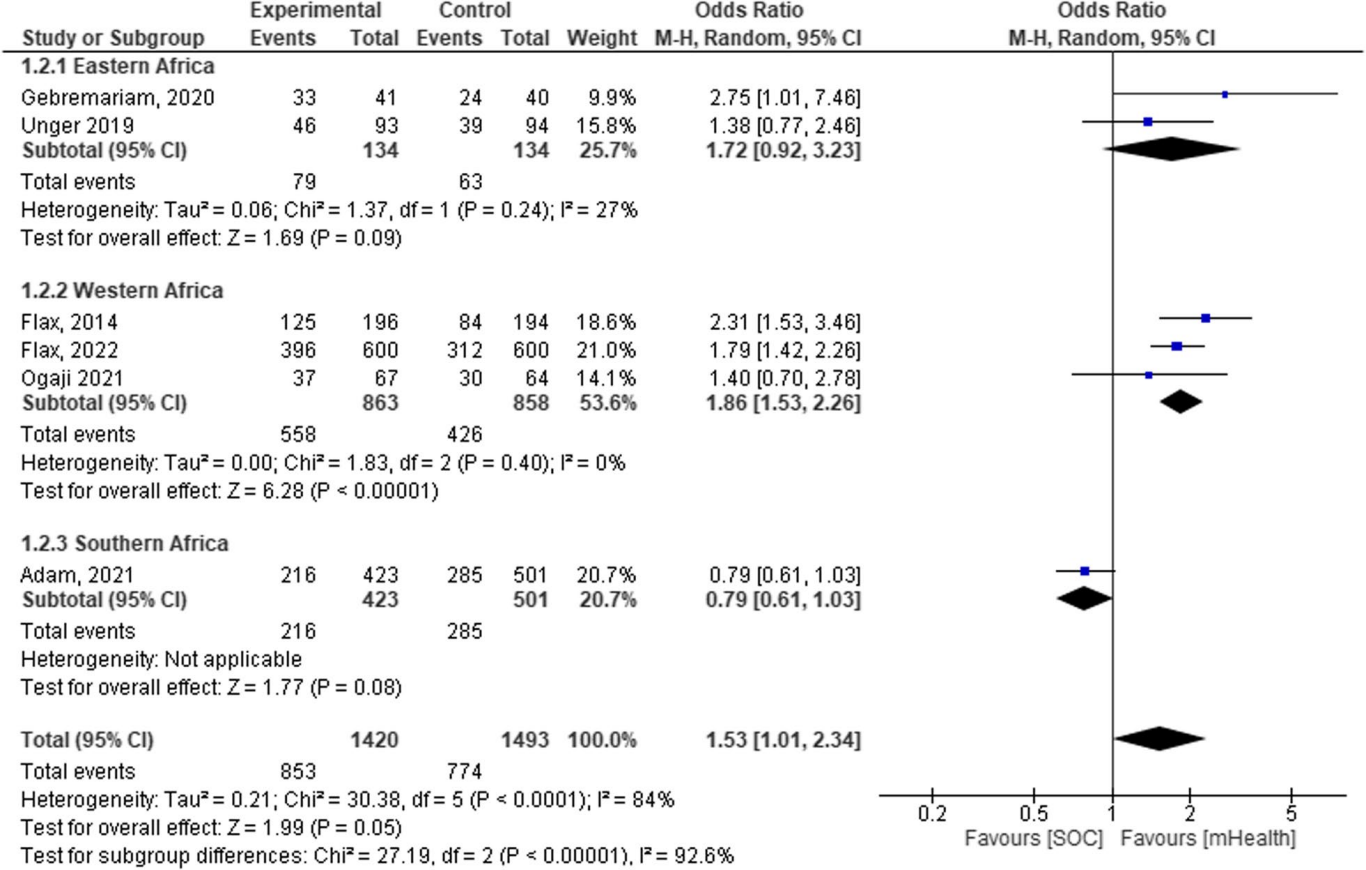
Forest plot showing the regional subgroup analysis in a systematic review and meta-analysis on child feeding practices in Africa

The mean effect size of Eastern, Western, and Southern African regions was 72% [OR = 1.72, 95% CI: 0.92–3.23; *P* < 0.09; I^2^ = 27%], 53% [OR = 1.86, 95% CI 1.53–2.26; *P* < 0.001; I^2^ = 0%], and 0.79% [OR = 0.79, 95% CI 0.61–1.03; *P* < 0.80] respectively. Thus, future studies and interventions need to account for regional variation [Fig. 10.]

### mHealth and mHealth plus additional supports

**Fig. 11:**
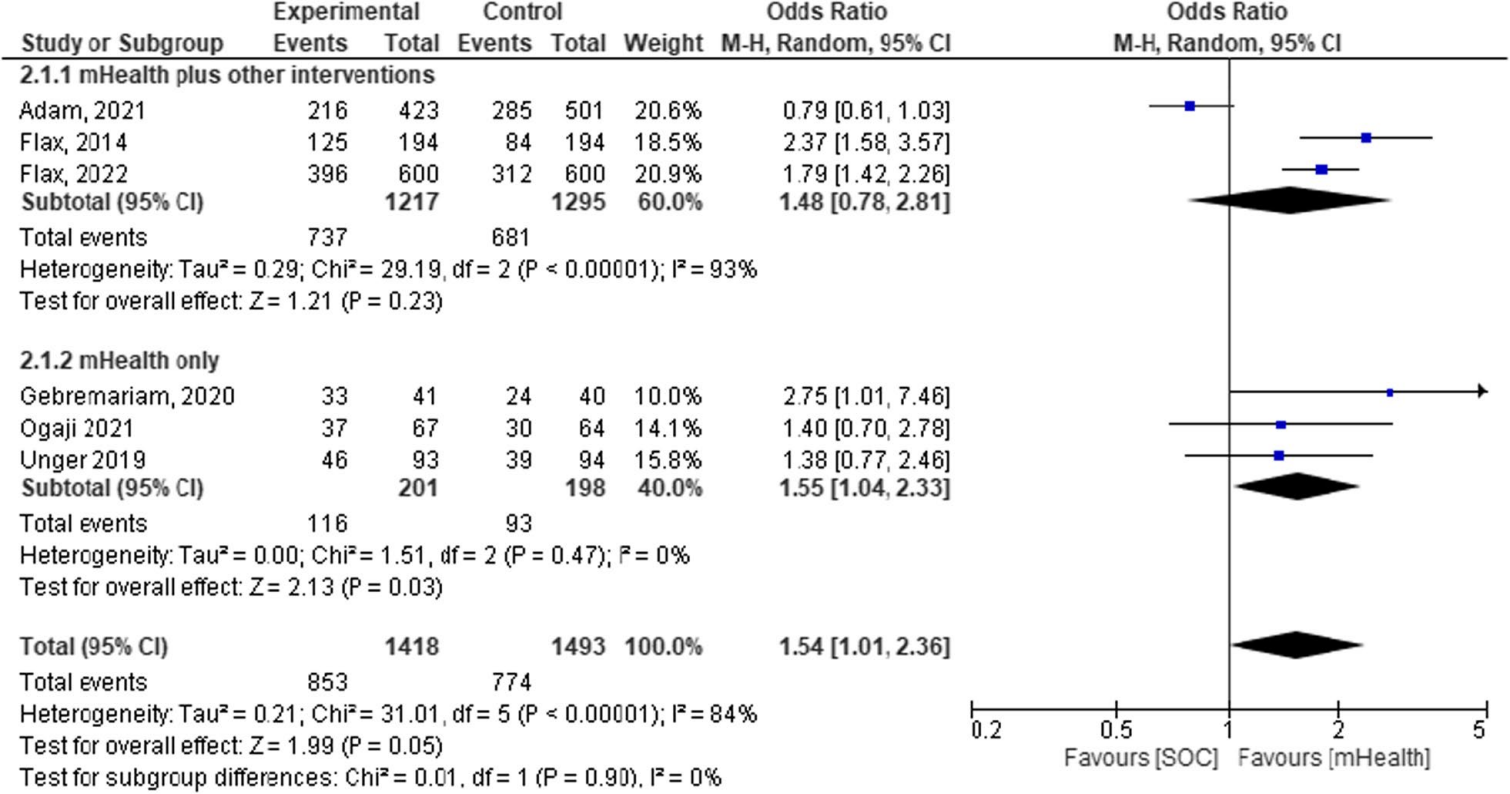
Forest plot depicting the subgroup analysis based on intervention in a systematic review and meta-analysis on child feeding practices in Africa

Mobile health plus additional supportive interventions showed a mean effect size of 48% [OR = 1.48, 95% CI 0.78–2.81; *P* = 0.23; I2 = 93%]. The finding has high heterogeneity. Compared to mHealth plus additional supportive interventions, the mHealth-only intervention was non-heterogeneous and uniform across the groups [OR = 1.55, 95% CI 1.04–2.33; *P* < 0.03; I2 = 0%]. However, the overall mean effect size remains uniform [OR = 1.54, 95% CI 1.01–2.36; *P* = 0.90; I^2^ = 0%] [Fig. 10] despite the two differences. The application of various additional supports in addition to mHealth across the studies might be the reason for the lack of uniformity (Fig. 11).

### Other analyses

#### EBF in the first month

**Fig. 12:**
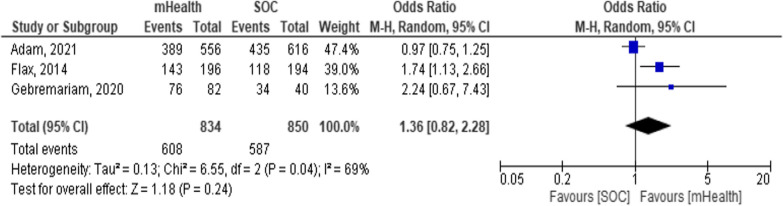
Forest plot showing EBF in the first month in a systematic review and meta-analysis on child feeding practices in Africa

Three studies [28, 41, 52] reported EBF in the first month after birth. There was no significant difference in EBF in the first month between groups [OR = 1.39, 95% CI 0.8–2.28; *P* = 0.24; I^2^ = 69%] [Fig. 10]. This might show that mHealth may only need to be planned for after the first month of birth (Fig. 12).

Generally, the included studies showed high heterogeneity although mHealth showed substantial improvement in child feeding practices. Application of subgroup analysis shows that regional variation and additional supportive interventions are the source of heterogeneity. The one study that identified influencing other studies has a larger sample size [weight]. The measurement of sensitivity and risk of bias showed that no study is over-influential and could be removed. Overall, the results show that mHealth is an important intervention to improve child-feeding practices in Africa.

## Discussion

Our systematic review and meta-analysis revealed that mHealth improves child-feeding problems in Africa [17, 30, 41, 42, 52, 53]. This means, mothers who are in mHealth intervention significantly improved their child feeding practice. This is an important finding because poor child feeding practice has been reported as a public health problem in other studies [15, 39]. The finding from this review is also consistent with other findings which reported improved breastfeeding efficacy and attitudes toward breastfeeding due to mHealth application [15, 43, 44].

Four studies [17, 30, 52, 53] have a small sample size (122–390) out of six and two studies [41, 42] have relatively large sample sizes (900 and 1200). The four studies with large sample sizes showed less significance in improving child-feeding practices compared to small sample-size studies. This evidence was not common in previous revisions [15, 43, 44]. However, this evidence is not enough to conclude the differences based on the sample size and we leave the space for further investigations. Of course, the presence of additional interventions might cause heterogeneity across studies and at least uniform application of additional intervention can improve comparability. Furthermore, it is also difficult to judge whether the studies with additional interventions have an advantage over those studies that messaged only health information to the participants based on this small number of studies. Thus, we invite further studies in a more uniform application of mHealth.

During subgroup analysis, we were able to rule out heterogeneity due to the sample size and design but the regions remained heterogeneous. This might be explained by the difference in socioeconomic development factors, education, a small number of studies, and another environmental status of the regions [15, 41]. Our subground analysis showed that one study from South Africa behaved differently and increased heterogeneity [41]. The South Africa study had a larger sample size but followed all required methodologies. The finding is interesting because if the mHealth effect decreases with an increased number of participants, it could give us clues for wider implementation. However, mHealth best worked in less developed regions [4] and the overall lower number of included studies does not provide enough evidence.

Our review included studies that assessed the effect of mHealth on breastfeeding in the first and fifth months since there are no other studies on child-feeding practices that fulfilled the inclusion criteria. Although the outcome in the fifth month after birth showed variation between mHealth and standard care, there was no difference between mHealth and standard care in the first month. The finding is consistent with individual and review studies [15, 41]. This might show that using mHealth to improve EBF can be planned for the period following the first month of birth. During the first month, all mothers might stay with their newborn and that might make breastfeeding similar among mothers of different status.

Our review has the following limitations. One study out of six was not published in a peer-reviewed journal [52]. This might raise questions about conclusions based on non-published evidence; however, missing available information because of the publication is also a bias. During meta-analysis, in two studies, there was evidence of maternal knowledge measured differently which made pooling maternal knowledge difficult. There were also studies with small sample sizes [52] or conducted in slightly different populations [30] that might raise the question of comparability. Additionally, the regional subgroup analysis might be affected by the number of studies per region. However, the authors followed standard guidelines for conducting meta-analysis and exhaustively searched the available databases for evidence to avoid bias. We examined the evidence carefully and identified subgroups. Additionally, funnel plots and other tests showed that no study can be over-influential. We also suggested a cautious use of the findings of this study in light of limitations.

## Conclusion

This systematic review and meta-analysis showed that mHealth improves child feeding in Africa. However, the heterogeneity is higher across the regions that need consideration when applying the findings. The included studies showed a moderate risk of bias because some studies had lower scores in participants and data collectors’ blinding. This moderately lowered the quality of evidence from our study. We also recommend studies to apply the seven Cochrane criteria for risk of bias (random sequence generation, allocation concealment, blinding of participants, blinding of outcome assessment, incomplete outcome data, selective reporting, and others) to obtain quality outcomes. Further studies on the mHealth effect on child feeding practice may need to consider regional variabilities, which might include socioeconomic development and socio-demographic characteristics differences. Studies might need to find common additional interventions with mHealth. The current finding provides policy direction for mHealth application and further studies to include additional interventions along with mHealth.

### Supplementary Information

**Additional file 1. **The search strategies containing eight accessed databases.

## Data Availability

This submission contains all the data used in the review.

## Abbreviations

WHO: World Health Organization
UNICEF: United Nations International Child Emergency Fund
OR: Odds ratio
USA: United States of America
EBF: Exclusive breast feeding

## References

[1] ENN, IFE Core Group. Operational guidance: breastfeeding counseling emergencies. Oncology. 2021. https://www.ennonline.net/breastfeedingcounsellinginemergencies.

[2] Wen LM, Rissel C, Xu H (2020). Effects of telephone and short message service support on infant feeding practices, “tummy time”, and screen time at 6 and 12 months of child age: a 3-group randomized clinical trial. JAMA Pediatr.

[3] Patel A, Kuhite P, Puranik A (2018). Effectiveness of weekly cell phone counselling calls and daily text messages to improve breastfeeding indicators. BMC Pediatr.

[4] Efrat MW, Esparza S, Mendelson SG, Lane CJ (2015). The effect of lactation educators implementing a telephone-based intervention among low-income Hispanics: a randomised trial. Health Educ J.

[5] Qian J, Wu T, Lv M, Fang Z, Chen M, Zeng Z (2021). The value of mobile health in improving breastfeeding outcomes among perinatal or postpartum women: systematic review and meta-analysis of randomized controlled trials. JMIR mHealth uHealth.

[6] Folaranmi T. mHealth in Africa: challenges and opportunities. vociecs. 2017. p. 1–6. https://www.bsg.ox.ac.uk/blog/mhealth-africa-challenges-and-opportunities. 10.1177/1757913913514703 24395839

[7] WHO, UNICEF, UNFPA. Maternal mortality. 2019. p. 1–5. https://www.who.int/news-room/fact-sheets/detail/maternal-mortality.

[8] World Health Organization. Complementary feeding: family foods for breastfed children. Dept Nutr Health Dev. 2000;1–56. https://motherchildnutrition.org.

[9] United Nations Children’s Fund (UNICEF). Improving young children’s diets during the complementary feeding period. UNICEF Programming Guidance. New York: UNICEF. 2020.

[10] Mbuthia F, Reid M, Fichardt A (2019). MHealth communication to strengthen postnatal care in rural areas: a systematic review. BMC Pregnancy Childbirth.

[11] Fadare O, Amare M, Mavrotas G, Akerele D, Ogunniyi A (2019). Mother’s nutrition-related knowledge and child nutrition outcomes: empirical evidence from Nigeria. PLoS ONE.

[12] Breastfeeding counselling in emergencies: operational guidance. Oncology. 2021 (3507. https://www.globalbreastfeedingcollective.org/media/1536/file/Breastfeeding-counselling-in-Emergencies-2021.pdf).

[13] Chai LK, Collins CE, May C, Brown LJ, Ashman A, Burrows TL (2021). Fidelity and acceptability of a family-focused technology-based telehealth nutrition intervention for child weight management. J Telemed Telecare.

[14] Khan SS, Patel A, Puranik A, Kuhite P, Pusdekar Y, Dibley MJ, Alam A (2020). Use of mobile health in infant and young child nutrition: a formative study in rural Maharashtra. Qual Rep.

[15] Qian J, Wu T, Lv M, Fang Z, Chen M, Zeng Z (2021). The value of mobile health in improving breastfeeding outcomes among perinatal or postpartum women: systematic review and meta-analysis of randomized controlled trials. JMIR mHealth uHealth.

[16] Jerin I, Akter M, Talukder K, Talukder MQEK, Rahman MA (2020). Mobile phone support to sustain exclusive breastfeeding in the community after hospital delivery and counseling: a quasi-experimental study. Int Breastfeed J.

[17] Ogaji DS, Arthur AO, George I (2021). Effectiveness of mobile phone-based support on exclusive breastfeeding and infant growth in Nigeria: a randomized controlled trial. J Trop Pediatr.

[18] Mekonnen ZA, Gelaye KA, Were MC, Tilahun B (2021). Acceptability, barriers and facilitators of mobile text message reminder system implementation in improving child vaccination: a qualitative study in northwest Ethiopia. J Multidiscip Healthc.

[19] Lee SH, Nurmatov UB, Nwaru BI, Mukherjee M, Grant L, Pagliari C. Effectiveness of mHealth interventions for maternal, newborn and child health in low- and middle-income countries: systematic review and meta-analysis. J Glob Health. 2016;6(1). 10.7189/jogh.06.010401 PMC4643860 26649177

[20] Jiang H, Li M, Wen LM, Hu Q, Yang D, He G (2014). Effect of short message service on infant feeding practice findings from a community-based study in Shanghai, China. JAMA Pediatr.

[21] Jiang H, Biviji R, Hmone MP, Li M, Alam A, Dibley MJ (2017). Mobile phone short messages to improve exclusive breastfeeding and reduce adverse infant feeding practices: protocol for a randomized controlled trial in Yangon, Myanmar. JMIR Res Protoc..

[22] Dol J, Richardson B, Tomblin Murphy G, Aston M, McMillan D, Campbell-Yeo M. Impact of mobile health (mHealth) interventions during the perinatal period for mothers in low- and middle-income countries: a systematic review. JBI Database System Rev Implement. 10.11124/JBISRIR-2017-004022 31404051

[23] Mangwi Ayiasi R, Kolsteren P, Batwala V, Criel BOC (2016). Effect of Village Health team home visits and mobile phone consultations on maternal and newborn care practices in Masindi and Kiryandongo, Uganda: a community-intervention trial. PLoS ONE.

[24] Zunza M, Cotton MF, Mbuagbaw L, Lester R, Thabane L (2017). Interactive weekly mobile phone text messaging plus motivational interviewing in promotion of breastfeeding among women living with HIV in South Africa : study protocol for a randomized controlled trial. Trials.

[25] Lakhani CM, Tierney BT, Jian AK, Pate M (2019). Short message service communication improves exclusive breastfeeding and early postpartum contraception in a low- to middle-income country setting: a randomised trial. Physiol Behav.

[26] Flax VL, Ibrahim AU, Negerie M, Yakubu D, Leatherman S, Bentley ME (2017). Group cell phones are feasible and acceptable for promoting optimal breastfeeding practices in a women’s microcredit program in Nigeria. Matern Child Nutr.

[27] Guyon A, Bock A, Buback L, Knittel B (2016). Mobile-based nutrition and child health monitoring to inform program development: an experience from Liberia. Glob Health Sci Pract.

[28] Flax VL, Ipadeola A, Schnefke CH, Ralph-Opara U, Adeola O, Edwards S (2022). Breastfeeding interpersonal communication, mobile phone support, and mass media messaging increase exclusive breastfeeding at 6 and 24 weeks among clients of private health facilities in Lagos, Nigeria. J Nutr.

[29] Achanyi-Fontem J (2013). Use of mobiles for breastfeeding counselling: the role of mobiles in promoting new learning. Pan-Commonwealth Forum.

[30] Flax VL, Negerie M, Ibrahim AU, Leatherman S, Daza EJ, Bentley ME (2014). Integrating group counseling, cell phone messaging, and participant-generated songs and dramas into a microcredit program increases Nigerian women’s adherence to international breastfeeding recommendations. J Nutr.

[31] Schneider L, Ollila S, Mutanen M (2022). The usefulness of nutrition and health videos displayed on mobile phones in rural Uganda: experiences of community health workers and mothers. Matern Child Nutr.

[32] Adam M, Johnston J, Job N, Dronavalli M, Le Roux I, Mbewu N (2021). Evaluation of a community-based mobile video breastfeeding intervention in Khayelitsha, South Africa: the Philani MOVIE cluster-randomized controlled trial. PLoS Med.

[33] Adam M, Tomlinson M, Le Roux I, Lefevre AE, McMahon SA, Johnston J (2019). The Philani MOVIE study: a cluster-randomized controlled trial of a mobile video entertainment-education intervention to promote exclusive breastfeeding in South Africa. BMC Health Serv Res.

[34] Media S. Research topics all publications methods short reads tools & resources experts abo. 2021; 2013:1–11. https://www.pewresearch.org/g.

[35] Royston G, Hagar C, Long LA, McMahon D, Pakenham-Walsh N, Wadhwani N (2015). Mobile health-care information for all: a global challenge. Lancet Glob Heal.

[36] Harris B, Ajisola M, Alam RM, Watkins JA, Arvanitis TN, Bakibinga P (2021). Mobile consulting as an option for delivering healthcare services in low-resource settings in low- and middle-income countries: a mixed-methods study. Digit Health.

[37] Sowon K, Maliwichi P, Chigona W (2022). The influence of design and implementation characteristics on the use of maternal mobile health interventions in Kenya: systematic literature review. JMIR mHealth uHealth.

[38] Obasola O. Using technology to promote safe maternal health practices in Nigeria | The AAS. 2022. p. 1–4. https://aasciences.africa/new.

[39] Ojo AI (2018). mHealth Interventions in South Africa: a review. SAGE Open.

[40] Shenoy A, Qashri A, Bothe M, Chifamba N, Downscaling VGI. Readiness for e-Health in Sub-Saharan Africa readiness for e-Health in Sub-Saharan Africa. 2020;(May).

[41] Adam M, Johnston J, Job N, Dronavalli M, Le Roux I, Mbewu N (2021). Evaluation of a community-aa mobile video breastfeeding intervention in Khayelitsha, South Africa: the Philani MOVIE cluster-randomized controlled trial. PLoS Med.

[42] Flax VL, Ipadeola A, Schnefke CH, Ralph-Opara U, Adeola O, Edwards S (2022). Breastfeeding interpersonal communication, mobile phone support, and mass media messaging increase exclusive breastfeeding at 6 and 24 weeks among clients of private health facilities in Lagos, Nigeria. J Nutr.

[43] Chen H, Chai Y, Dong L, Niu W, Zhang P (2018). Effectiveness and appropriateness of mHealth interventions for maternal and child health: systematic review. JMIR mHealth uHealth.

[44] Lau Y, Htun TP, Tam WSW, Klainin-Yobas P (2016). Efficacy of e-technologies in improving breastfeeding outcomes among perinatal women: a meta-analysis. Matern Child Nutr.

[45] Tufanaru C, Munn Z, Aromataris E, Campbell J, Hopp L. JBI critical appraisal checklist for randomized controlled trials. Chapter 3: systematic reviews of effectiveness. 2020. p. 3. https://synthesismanual.jbi.global.

[46] Review Manager (RevMan) [Computer program]. Version 5.4.1. The Cochrane Collaboration. 2020.

[47] Higgins JPT, Thompson SG, Deeks JJ, Altman DG (2003). Measuring inconsistency in meta-analyses. BMJ.

[48] Borenstein M, Hedges LV, Higgins JPT, Rothstein HR, Borenstein M, Hedges LV, Higgins JPT, Rothstein HR (2009). Front matter. Introduction to meta-analysis.

[49] Egger M, Smith GD, Schneider M, Minder C (1997). Bias in meta-analysis detected by a simple, graphical test. Br Med J.

[50] Duval S, Tweedie R (2000). Trim and fill: A simple funnel-plot-based method of testing and adjusting for publication bias in meta-analysis. Biometrics.

[51] Kalan R, Wiysonge CS, Ramafuthole T, Allie K, Ebrahim F, Engel ME (2014). Mobile phone text messaging for improving the uptake of vaccinations: a systematic review protocol. BMJ Open.

[52] Gebremariam KT. Effectiveness of SMS text messaging to improve exclusive breastfeeding in Mekelle, Ethiopia. 2020;160–192. https://eprints.qut.edu.a. https://eprints.qut.edu.au/205813/1/KidaneTadesse_Gebremariam_Thesis.pdf

[53] Unger JA, Ronen K, Perrier T, DeRenz IB, Slyker J, Drake AL, Mogaka D, Kinuthia JJ-SG (2018). SMS communication improves exclusive breastfeeding and early postpartum contraception in a low to middle income country setting: a randomised trial. BJOG.

