# Supplementary material for: Assessing the effect of mHealth on child feeding practice in African countries: systematic and meta-analysis
Source: J Health Popul Nutr. 2023 Dec 8;42:138. doi: 10.1186/s41043-023-00487-y (PMC10704613; doi:10.1186/s41043-023-00487-y)
Supplement: Supplementary file 1 — Additional file 1. The search strategies containing eight accessed databases. [file 41043_2023_487_MOESM1_ESM.docx]

We applied different search strategies to find available materials. Since our study includes both published and unpublished literature, we used databases like PubMed, Cochrane Library, Web of Science, EMBASE, ClinicalTrials.gov, Sciencedirect, African Journals Online (AJOL), AND WHO International Clinical Trials Registry Platform (ICTRP) Google Scholar, Global Health, HINARI, and CINAR for peer-reviewed studies. For the unpublished studies, we used the most-read web portals like WHO, mHealth Alliance, arxiv, AAS Open Research, Advance: a SAGE Preprints Community, Research gate, Research square AfricArxiv, AMRC Open Research, etc., and International Development Research Center reports and Profit-based and nonprofit organizational websites

**1. PubMed**

“exclusive breastfeeding”[title/abstract] OR “breastfeeding”[title/abstract] OR ”prelacteal feeding”[title/abstract] OR ”initiation of breastfeeding”[title/abstract] OR ”bottle feeding”[title/abstract] OR ”timely complementary feeding”[title/abstract] OR ”complementary feeding”[title/abstract]

#8“Mobile Applications”[MESH]

#9“Telemedicine”[MESH]

#10“Text Messaging”[MESH]

#11“Cell Phone”[MESH]

#12“Smartphone”[MESH]

#13“Mobile Applications” OR “mobile” OR “Telemedicine” OR “telephone” OR “Text Messaging” OR "Telenursing" OR “Cell Phone” “cellular phone” OR “phone” OR tablet* OR ipad* OR ipod* OR android OR nexus OR iOS OR handheld* OR hand-held* OR notebook* OR "note book*" OR “multimedia message” OR “multi-media message” OR “Smartphone” OR “smart-phone” OR “mHealth” OR “Mobile” OR “Portable Software Application” OR “Tele*” OR “m-Health” OR “?phone*” OR “Text*” OR “Short Message” OR “SMS” OR “App” OR “Apps” OR “App-based” OR “Electronic” OR “Message*” OR “Web” OR “Web-based” OR “Internet*” OR “Digital*”

#14(randomized controlled trial[pt] OR controlled clinical trial[pt] OR randomized[tiab] OR placebo[tiab] OR clinical trials as topic[mesh:noexp] OR randomly[tiab] OR trial[ti]) NOT (animals [mh] NOT (humans [mh] AND animals[mh]))

#15([Algeria](http://listofafricancountries.com/Algeria) OR [Angola](http://listofafricancountries.com/Angola) OR [Benin](http://listofafricancountries.com/Benin) OR [Botswana](http://listofafricancountries.com/Botswana) OR [Burkina Faso](http://listofafricancountries.com/Burkina+Faso) OR [Burundi](http://listofafricancountries.com/Burundi) OR [Cameroon](http://listofafricancountries.com/Cameroon) OR [Canary Islands](http://listofafricancountries.com/Canary+Islands) OR [Cape Verde](http://listofafricancountries.com/Cape+Verde) OR [Central African Republic](http://listofafricancountries.com/Central+African+Republic) OR [Ceuta](http://listofafricancountries.com/Ceuta) OR [Chad](http://listofafricancountries.com/Chad) OR [Comoros](http://listofafricancountries.com/Comoros) OR [Côte d'Ivoire](http://listofafricancountries.com/C%C3%B4te+d%27Ivoire) OR [Democratic Republic of the Congo](http://listofafricancountries.com/Democratic+Republic+of+the+Congo) OR [Djibouti](http://listofafricancountries.com/Djibouti) OR [Egypt](http://listofafricancountries.com/Egypt) OR [Equatorial Guinea](http://listofafricancountries.com/Equatorial+Guinea) OR [Eritrea](http://listofafricancountries.com/Eritrea) OR [Ethiopia](http://listofafricancountries.com/Ethiopia) OR [Gabon](http://listofafricancountries.com/Gabon) OR [Gambia](http://listofafricancountries.com/Gambia) OR [Ghana](http://listofafricancountries.com/Ghana) OR [Guinea](http://listofafricancountries.com/Guinea) OR [Guinea-Bissau](http://listofafricancountries.com/Guinea-Bissau) OR [Kenya](http://listofafricancountries.com/Kenya) OR [Lesotho](http://listofafricancountries.com/Lesotho) OR [Liberia](http://listofafricancountries.com/Liberia) OR [Libya](http://listofafricancountries.com/Libya) OR [Madagascar](http://listofafricancountries.com/Madagascar) OR [Madeira](http://listofafricancountries.com/Madeira) OR [Malawi](http://listofafricancountries.com/Malawi) OR [Mali](http://listofafricancountries.com/Mali) OR [Mauritania](http://listofafricancountries.com/Mauritania) OR [Mauritius](http://listofafricancountries.com/Mauritius) OR [Mayotte](http://listofafricancountries.com/Mayotte) OR [Melilla](http://listofafricancountries.com/Melilla) OR [Morocco](http://listofafricancountries.com/Morocco) OR [Mozambique](http://listofafricancountries.com/Mozambique) OR [Namibia](http://listofafricancountries.com/Namibia) OR [Niger](http://listofafricancountries.com/Niger) OR [Nigeria](http://listofafricancountries.com/Nigeria) OR [Republic of the Congo](http://listofafricancountries.com/Republic+of+the+Congo) OR [Réunion](http://listofafricancountries.com/R%C3%A9union) OR [Rwanda](http://listofafricancountries.com/Rwanda) OR [Saint Helena](http://listofafricancountries.com/Saint+Helena) OR [São Tomé and Príncipe](http://listofafricancountries.com/S%C3%A3o+Tom%C3%A9+and+Pr%C3%ADncipe) OR [Senegal](http://listofafricancountries.com/Senegal) OR [Seychelles](http://listofafricancountries.com/Seychelles) OR [Sierra Leone](http://listofafricancountries.com/Sierra+Leone) OR [Somalia](http://listofafricancountries.com/Somalia) OR [South Africa](http://listofafricancountries.com/South+Africa) OR [Sudan](http://listofafricancountries.com/Sudan) OR [Swaziland](http://listofafricancountries.com/Swaziland) OR [Tanzania](http://listofafricancountries.com/Tanzania) OR [Togo](http://listofafricancountries.com/Togo) OR [Tunisia](http://listofafricancountries.com/Tunisia) OR [Uganda](http://listofafricancountries.com/Uganda) OR [Western Sahara](http://listofafricancountries.com/Western+Sahara) OR [Zambia](http://listofafricancountries.com/Zambia) OR [Zimbabwe](http://listofafricancountries.com/Zimbabwe)

)

#16 #1 OR #2 OR #3 OR #4 OR #5 OR #6 OR #7

#17 #8 OR #9 OR #10 OR #11 OR #12 OR #13

#18 #15

#19 #14 AND #16 AND #17 AND #18

**2. Web of Science**

#1“breastfeeding” OR “exclusive breastfeeding” OR “initiation of breastfeeding” OR ”prelacteal feeding” OR “timing of complementary feeding” OR “complementary feeding” OR “bottle feeding”

#2“Mobile Applications” OR “Telemedicine” OR “Text Messaging” OR “Cell Phone” OR “Smartphone” OR “mobile” OR “Portable Software Application” OR “Tele*” OR “mHealth” OR “m-Health” OR “?phone*” OR “Text*” OR “Short Message” OR “SMS” OR “app” OR “apps” OR “app-based” OR “electronic” OR “Message*” OR “web” OR “web-based” OR “Internet*” OR “digital*”

#3 TS= clinical trial* OR TS=research design OR TS=comparative stud* OR TS=evaluation stud* OR TS=controlled trial* OR TS=follow-up stud* OR TS=prospective stud* OR TS=random* OR TS=placebo* OR TS=(single blind*) OR TS=(double blind*)

**3. Cochrane Library**

##1“exclusive breastfeeding”

#2“breastfeeding”

#3”prelacteal feeding”

#4”initiation of breastfeeding”

#5”bottle feeding”

#6”timely complementary feeding”

#7”complementary feeding”

#8breastfeeding[MESH descriptor]

#9 “Mobile Applications”[MESH descriptor]

#10 Telemedicine[MESH descriptor]

#11 “Text Messaging”[MESH descriptor]

#12 “Cell Phone”[MESH descriptor]

#13 Smartphone[MESH descriptor]

#14 mobile OR “Portable Software Application” OR Tele* OR mHealth OR eHealth OR e-health OR m-Health OR ?phone* OR Text* OR “Short Message” OR SMS OR app OR apps OR “app-based” OR electronic OR Message* OR web OR “web-based” OR Internet* OR digital*

#15 #1OR #2 OR #3 OR #4 OR #5 OR #6 OR #7 OR #8

#16 #9 #10 OR #11 OR #12 OR #13 #14 OR

#17 #15 AND #16

**4. Embase**

#1'breastfeeding'/exp OR 'exclusive breastfeeding'/exp OR ‘exclusive breastfeeding’/exp OR ‘breastfeeding’/exp OR ‘prelacteal feeding’/exp OR ‘initiation of breastfeeding’/exp OR ‘bottle feeding’/exp OR ‘timely complementary feeding’/exp OR ‘complementary feeding’/exp

#2'mobile application'/exp OR 'telemedicine'/exp OR 'text messaging'/exp OR 'mobile phone'/exp OR 'smartphone'/exp

#3'mobile':ti,ab,kw OR 'portable software application':ti,ab,kw OR 'tele*':ti,ab,kw OR 'mhealth':ti,ab,kw OR 'm-health':ti,ab,kw OR '?phone*':ti,ab,kw OR 'text*':ti,ab,kw OR 'short message':ti,ab,kw OR 'sms':ti,ab,kw OR 'app':ti,ab,kw OR 'apps':ti,ab,kw OR 'app-based':ti,ab,kw OR 'electronic':ti,ab,kw OR 'message*':ti,ab,kw OR 'web':ti,ab,kw OR 'web-based':ti,ab,kw OR 'internet*':ti,ab,kw OR 'digital*':ti,ab,kw

#4 ('crossover procedure':de OR 'double-blind procedure':de OR 'randomized controlled trial':de) AND or  AND 'single-blind procedure':de OR (random*:de,ab,ti AND or :de,ab,ti AND factorial*:de,ab,ti) OR crossover*:de,ab,ti OR ((cross NEXT/1 over*):de,ab,ti) OR placebo*:de,ab,ti OR ((doubl* NEAR/1 blind*):de,ab,ti) OR ((singl* NEAR/1 blind*):de,ab,ti) OR assign*:de,ab,ti OR allocat*:de,ab,ti OR volunteer*:de,ab,ti AND Africa

#5 #2 OR #3

#6 #1 AND #4

#7 #5 AND #6

**5. ClinicalTrials.gov**

mHealth OR digital OR Mobile OR Smartphone OR "Cell phone" OR Techno OR "short message service" OR SMS OR Tele OR Telemedicine OR Telehealth OR E-health OR eHealth OR Remote OR Electro OR Comput OR cloud OR Software OR Application AND breastfeeding OR early breastfeeding OR complementary feeding OR timely complementary feeding OR prelacteal feeding AND Africa

**6. Sciencedirect**

mHealth OR Mobile OR Smartphone OR "Cell phone" OR Techno OR "short message service" OR SMS OR Tele OR Telemedicine OR Telehealth OR E-health OR eHealth OR Remote OR Electro OR Comput AND breastfeeding OR early breastfeeding OR complementary feeding OR timely complementary feeding OR prelacteal feeding AND Africa

**7. African Journals Online (AJOL)**

mHealth OR Mobile OR Smartphone OR "Cell phone" OR Techno OR "short message service" OR SMS OR Tele OR Telemedicine OR Telehealth OR E-health OR eHealth OR Remote OR Electro OR Comput AND breastfeeding OR early breastfeeding OR complementary feeding OR timely complementary feeding OR prelacteal feeding AND Africa

**8. WHO International Clinical Trials Registry Platform (ICTRP)**

mHealth OR Mobile OR Smartphone OR "Cell phone" OR Techno OR "short message service" OR SMS OR Tele OR Telemedicine OR Telehealth OR E-health OR eHealth OR Remote OR Electro OR Comput AND breastfeeding OR early breastfeeding OR complementary feeding OR timely complementary feeding OR prelacteal feeding AND Africa
